# Macrophages and Associated Ligands in the Aged Injured Nerve: A Defective Dynamic That Contributes to Reduced Axonal Regrowth

**DOI:** 10.3389/fnagi.2020.00174

**Published:** 2020-06-12

**Authors:** Jo Anne Stratton, Shane Eaton, Nicole L. Rosin, Sana Jawad, Alexandra Holmes, Grace Yoon, Rajiv Midha, Jeff Biernaskie

**Affiliations:** ^1^Hotchkiss Brain Institute, University of Calgary, Calgary, AB, Canada; ^2^Department of Comparative Biology and Experimental Medicine, Faculty of Veterinary Medicine, University of Calgary, Calgary, AB, Canada; ^3^Alberta Children’s Hospital Research Institute, University of Calgary, Calgary, AB, Canada; ^4^Department of Clinical Neuroscience, Faculty of Medicine, University of Calgary, Calgary, AB, Canada

**Keywords:** aging, nerve injury, macrophages, microenvironment, MCP1, axonal regeneration

## Abstract

The regenerative capacity of injured peripheral nerves is diminished with aging. To identify factors that contribute to this impairment, we compared the immune cell response in young vs. aged animals following nerve injury. First, we confirmed that macrophage accumulation is delayed in aged injured nerves which is due to defects in monocyte migration as a result of defects in site-specific recruitment signals in the aged nerve. Interestingly, impairment in both macrophage accumulation and functional recovery could be overcome by transplanting bone marrow from aged animals into young mice. That is, upon exposure to a youthful environment, monocytes/macrophages originating from the aged bone marrow behaved similarly to young cells. Transcriptional profiling of aged macrophages following nerve injury revealed that both pro- and anti-inflammatory genes were largely downregulated in aged compared to young macrophages. One ligand of particular interest was macrophage-associated secreted protein (MCP1), which exhibited a potent role in regulating aged axonal regrowth *in vitro*. Given that macrophage-derived MCP1 is significantly diminished in the aged injured nerve, our data suggest that age-associated defects in MCP1 signaling could contribute to the regenerative deficits that occur in the aged nervous system.

## Introduction

The mammalian PNS can regenerate following nerve injury, but the extent of recovery depends on several factors, including age; where age is negatively correlated with regeneration (Huebner and Strittmatter, 2009). Indeed, both axon regrowth and behavioral recovery are diminished in aged animals relative to young animals following an equivalent nervous injury (Pestronk et al., 1980; Tanaka and Webster, 1991; Vaughan, 1992; Verdú et al., 2000). The mechanisms underlying this age-related neuro-regenerative deficiency remains unknown. Effective treatments that target the aging population to enhance recovery following nerve injury do not exist.

Previous work has demonstrated that aged axons retain their capacity for regrowth, equivalent to young axons, given the appropriate environment (Painter et al., 2014; Scheib and Höke, 2016). This suggests that the surrounding injury environment within the aged nerve contributes to the suppression of regeneration. Secreted factors from macrophages—located in regions distal to the injury site—can directly affect axonal regrowth (Kigerl et al., 2009). Depending on macrophage phenotypes, axonal regrowth can be either enhanced or suppressed. Others have provided a thorough characterization of nerve macrophages as well as demonstrated the importance of macrophages by preventing their accumulation following nerve injury (Tanaka and Webster, 1991; Barrette et al., 2008; Stratton et al., 2018; Ydens et al., 2020). This revealed that the number of regenerating nerve fibers and the extent of functional recovery is significantly reduced in mice where macrophage accumulation is inhibited.

Here, we sought to explore macrophage dynamics that are associated with defective axonal regeneration in aged mice. Specifically, we investigated changes in macrophage dynamics as a potential contributor to the loss of regenerative competence within the aged nerve. We discovered that the injured microenvironment within the aged nerve plays a critical role in shaping macrophage dynamics. Also, we demonstrated that the number of macrophages expressing Chemokine (C-C motif) ligand 2 (CCL2/MCP1) was reduced following nerve injury in aged mice and that exposing aged axons to MCP1 was able to significantly enhance their regrowth (Niemi et al., 2016). This data could suggest that reduced MCP1 signaling might contribute to the regenerative deficiency observed in the aged nervous system.

## Materials and Methods

### Rodent Nerve Injuries

All experiments using animals were carried out following the Canadian Council of Animal Care Guidelines, and approved by the University of Calgary’s Health Sciences Animal Care Committee. All surgeries and electrophysiological readings were carried out under isoflurane (2%–5%) inhalation anesthetic with postoperative pain control provided by intraperitoneal injections of buprenorphine (0.05 mg/kg) on the day of surgery, followed by buprenorphine on the days following. All surgical and electrophysiological procedures were carried out following the shaving of the hind limb area in an aseptic fashion using 70% ethanol. Under operating microscopes (M651, Leica), one sciatic nerve per animal was exposed at the sciatic notch and crushed for 10–20 s using beveled Dumont No. 5 forceps (L4$\frac{1}{4}$inch, Inox alloy, Sigma).

### Mice

Mice were maintained at the University of Calgary’s Animal Facility, under standard housing conditions. This included housing with 4–5 mice under a 12-h light-dark cycle, at 20–24°C, with unlimited food and water. *Cx3cr1-GFP/Ccr2-RFP* mice on a *C57BL/6* background (stock no.: 017586; stock No.: 005582, Jackson Laboratories), were kindly donated by Dr. Kubes (University of Calgary) in 2014. Mice were maintained *via* crossing *Cx3cr1-GFP^+/+^* *Ccr2-RFP^+/+^* male and *C57BL/6* female mice to obtain *Cx3cr1-GFP^+/–^* *Ccr2-RFP^+/–^* heterozygous offspring where both monocytes and macrophages were confirmed to have GFP and RFP reporters (Dal-Secco et al., 2015). *Sox2^tm2HochGFP^* mice were maintained on a mixed background (The Jackson Laboratory). Young mice were aged 3–6 months, and aged mice were 16–24 months old.

### CMAPs

Compound muscle action potential (CMAP) amplitudes were measured at 4, 6, and 8-weeks post-injury from uninjured and injured nerves, with the average value recorded from three measurements at the same stimulation intensity. The body temperature of animals was kept constant at 37°C ± 0.5°C throughout the experiment on a heating pad. The sciatic nerve was stimulated just above the sciatic notch using bipolar hook electrodes and the electromyogram activity was recorded (100×; 100 Hz to 1 kHz) using bipolar recording electrodes inserted into the gastrocnemius muscle of the corresponding hind limb. The experimenter was blinded to the post-injury time during recording.

### Dorsal Root Ganglion (DRG) Neuronal Cell Culture

Dorsal root ganglions (DRGs) were dissected out, trimmed of nerve roots and connective tissue, and washed in L15 medium. The cleaned DRGs were incubated in 0.1% collagenase IV/L15 at 37°C for 60 min, then dissociated by pipette trituration. The resulting cell suspension was centrifuged at 100 *g* for 6 min, and the pellet resuspended in L15 medium for density gradient filtration: the suspension was carefully laid over a 15% BSA/L15 solution and centrifuged as above, followed by a final L15 resuspension and centrifugation. The final pellet of purified DRG neurons was resuspended in DMEM/F12 media and plated on PDL/laminin-coated 96-well plate at a density of 2,500 cells/mL. Growth media consisted of DMEM/F12 enriched with 1× N2 supplement, 50 U/ml penicillin-streptomycin, 0.1% BSA, and 0.1 ng/ml NGF. In each well, either MCP1 (500 ng/ml), NGF (100 ng/ml), Il6 (500 ng/ml), or Il1β (50 ng/ml) were added as per experimental conditions, with PBS used as a control. The plated neurons were allowed to grow for 18–24 h at 37°C before being fixed in 4% PFA/1× PHEM and washed in 1× PBS. Cell culture experiments were performed in triplicates and were repeated at least three times on independent days.

### Immunocytochemistry

Fixed DRG neurons were incubated in blocking solution (5% donkey serum, 0.3% Triton X-100/1× PBS) for 30 min, primary antibodies in diluent (0.1% Triton X-100, 0.1% BSA, 0.04% EDTA/1× PBS) for 1 h, washed twice in PBS, secondary antibodies in diluent for 1 h, Hoechst-33258 (14530, Sigma, 1:500 in PBS) for 10 min, and finally washed twice in PBS. All steps were performed at RT. Primary antibodies used were a cocktail of anti-Neurofilament200 (mouse, N5389, Sigma-Aldrich, 1:800) and anti-Tubulin β3 (mouse, 801213, Bio Legend, 1:1,000) to facilitate automated neurite analysis. The secondary antibody used was Alexa Fluor 488 donkey anti-mouse (A21202, Invitrogen, 1:200). Each well was imaged at 10× using the ImageXpress system (Molecular Devices) and a composite was stitched using Microsoft Image Composite Editor (Microsoft). The composite images were each divided into nine images of equal size and all analyzed using the MetaXpress (Molecular Devices) automated neurite outgrowth function.

### Immunohistochemistry

Before tissue collection, mice were euthanized using CO_2_ asphyxiation. Dissected nerves were post-fixed in 4% PFA at 4°C overnight and subsequently allowed to equilibrate in 30% sucrose at 4°C overnight, before embedding in cryoprotectant (VWR Clear Frozen Section Compound) for storage at −80°C. Sciatic nerve segments stretching from 2 mm proximal to 1 cm distal from the crash site were collected. Twelve micron thick longitudinal sections were cut and stored at −80°C. Two to three high-quality sections per nerve per condition were selected for immunohistochemistry. Briefly, sections were permeabilized and blocked in 0.3% Triton X-100 and 5% BSA (1+ hour at room temperature) before primary antibody incubation overnight at room temperature. Immunostains were done using anti-ionized calcium-binding adaptor molecule 1 (rabbit, Wako, 1:500), Anti-Murine JE/MCP-1 (rabbit, Peprotech, 1:500), anti-Stathmin-2 (rabbit, NBP1-49461, Novus Biologicals, 1:1,000), Purified anti-Tubulin β3 (mouse, 801213, BioLegend, 1:1,000). After washing, cells were incubated at room temperature for 1–2 h with secondary antibodies conjugated to either Alexa Fluor 488, 555 or 647 (donkey anti-mouse, rabbit, rat, goat, 1:200, Invitrogen), and Hoechst-33258 (1:1,000, 14530, Sigma), then washed and cover-slipped. All immunohistochemical (IHC) stains were confirmed positive with appropriate no primary (secondary alone) controls. Image collection and quantification was done using either Nikon A1 or a Leica SP8 confocal microscope. Whole nerve montages were collected using a 63× objective lens, Tile-Scan and Z-stack features, and maximum projection images. For all other images, sections were imaged at 63× objective, 10–20 serial z-stacks in 0.5–1.0 μm steps, then maximum projected before quantifying. Three images per animal were collected from the crash site. All imaging of a given stain was performed using the same laser settings. We used ImageJ and designated markers to count cells or cellular components of interest, as well as nuclear stains. All quantification was done while blinded.

### Monocyte Migration Assay

Blood was collected from young and aged mice (*n* = 3 each) and centrifuged for 15 min at 300 *g*. The buffy coat was collected and the red blood cells were lysed. Using HBSS with 0.1% BSA, the cells were resuspended (1 × 10^6^ blood cells) and plated in the bottom of migration assay wells with 0.6 ml of HBSS with 0.1% BSA and 1/3 of the nerve section dissected into 1 mm pieces. The control wells contained 5% FBS. All wells were topped with 100 μl of cell suspension (1 × 10^5^ cells/well). Four hours later, the cells were collected from the bottom of the wells and analyzed using FACS sorting. The membranes were collected, fixed, and coverslipped with permount. The nerve tissue and supernatant from the bottom of the wells were collected and stored at −80°C.

### Bone Marrow Transplants

The recipient mice were lethally irradiated with a dosing schedule of 2 × 5.5 Gy, 4 h apart using a gamma irradiator with a cesium-137 source (Gammacell 40; Best Theratronics). Donors were euthanized and the femurs isolated on ice. The bone marrow was flushed from the femurs with 10 ml of HBSS and then centrifuged for 15 min at 300 *g*. The red blood cells were lysed and bone marrow cells resuspended at 5 × 10^7^ bone marrow cells/ml in HBSS. Two-hundred microliter (10 × 10^6^ bone marrow cells) were injected *via* the tail vein of prepared recipients. Recipient mice were used for experimentation at 8 weeks post-transplant.

### Macrophage FACs Isolations and RNAseq

At Day 3 and 8 post-nerve injury, *Cx3cr1-*GFP^+^* Ccr2-*RFP^+^ double-positive cells were FACS collected from 5 months (young) and 18 months (aged) *Cx3cr1*^GFP^/*Ccr2*^RFP^ mice. Following euthanasia, sciatic nerves were isolated, sterilized, and cleared of hair, membranes, and connective tissue. Nerves were finely chopped using a sterile blade, then put in collagenase (2 mg/ml, Worthington, Lakewood, NJ, USA) at 37°C for 30 min, triturating every 10 min until solutions appeared cloudy, then filtered with a 70 μm filter. Samples were centrifuged for 5 min at 200 *g*, the supernatant discarded, then re-suspended in 0.5% BSA in HBSS before FACS purification using a BD FACS Aria machine. A stringent initial gate was used to exclude debris and cell doublets. Wild-type cells were used as negative controls to determine gates for detecting GFP and RFP, and then macrophages were purified according to these gates. The number of cells collected from each animal is listed: Day 3 young (*n* = 7)—19,041, 13,548, 23,989, 8,621, 16,751, 8,621, 16,751; Day 3 aged (*n* = 7)—8,472, 11,194, 5,044, 2,338, 2,928, 2,338, 2,928. Due to low levels of RNA, samples were pooled so two samples per condition were analyzed. Total RNA ranged from 153 to 261 ng per pooled sample. All cells were sorted directly into lysis buffer (Ambion), then triturated and vortexed before storage at −80°C. For RNA extractions, all samples were processed together following manufacturers’ recommendations (AM1561, Ambion). Total RNA extracts were quantitated using a Qubit Fluorimeter and the Qubit HS assay kit (Invitrogen). All samples were subjected to reverse transcription and Multiple Displacement Amplification (MDA) with REPLI-g SensiPhi DNA Polymerase and oligo-dT primers as per the Qiagen REPLI-g Single Cell RNA Library preparation kit (150073) and the manufacturer’s protocol. The amplified cDNA product was then sheared to an average of 300 bp using a Covaris S220 sonicator and Covaris microtubes with AFA fibers (520045). Illumina compatible libraries were then prepared using the Qiagen GeneRead Adapter I Set A 12-plex (180985) index adapters as per the REPLI-g single-cell RNA library prep kit’s protocol. Products were quantitated and sized using both a Qubit fluorimeter with QuantiFluor dsDNA System dye (Promega E2670) and an Agilent 2200 TapeStation with D1000 ScreenTape and reagents (5067–5583). Five hundred to six hundred nanogram of the final library was obtained from each sample. Each library was quantitated using the Kapa qPCR Library Quantitation Kit for Illumina (KK4835) on a StepOne Plus qPCR instrument before preparing a single pool containing equal amounts of each library. This pool was then subjected to on-board cluster formation and sequencing on an Illumina NextSeq 500 sequencer with a high-output v2 75 cycle sequencing kit (FC-404-2005) as per the standard Illumina protocols. After sequencing, the bcl data was converted to fastq data files using the Illumina BCL2FASTQ utility. The sequencing run produced 475 million clusters passing filter (density = 206 K/mm^2^) and 34.8 gigabases of sequencing with quality scores of ≥Q30 (91.4%). The number of assigned reads for each sample was between 39.1 million and 43.9 million. Reads were pseudoaligned to the mouse NCBI RefSeq transcript database (O’Leary et al., 2016) dated January 2017, using Kallisto 0.42.4 (Bray et al., 2016). Sleuth (Pimentel et al., 2017) was used for differential gene-level expression using a linear model containing one term: the nominal age factor. Genes passing the Wald Test with Benjamini-Hochberg corrected *p*-values (a.k.a. false discovery rate, FDR) less than 0.05 were considered differentially expressed. Differentially expressed genes were annotated and analyzed for enrichment using Ingenuity Pathway Analysis (Qiagen N.V., Redwood City, CA, USA).

### Quantification and Statistical Analysis

For comparisons of groups across multiple groups, a one-way analysis of variance (ANOVA) followed by Tukey’s *post hoc* comparison was used (GraphPad Prism 5). For comparisons of groups across two groups, an unpaired Student *t*-test was used. For comparisons of multiple factors across two groups, multiple unpaired Student *t*-tests with a false discovery rate of 5% were used. *P*-values < 0.05 was considered significant. All other statistical details can be found in figure legends and “Materials and Methods” section.

## Results

### Diminished Axonal Regeneration in Aged Rodents Is Associated With the Aged Microenvironment

To confirm that axonal regeneration is defective in aged mice, we compared CMAP amplitudes in muscles innervated by previously injured nerves (Supplementary Figure S1A). This revealed that following a nerve crush injury, CMAP amplitudes were reduced to 9.06 ± 6.48% in the young group and 8.96 ± 4.71% (of uninjured) in the aged group at 4 weeks post-injury (Supplementary Figure S1B). Consistent with the literature (Verdú et al., 1995), by 8 weeks this had recovered to 52.34 ± 13.36% in young mice and only 33.84 ± 8.49% in aged mice (Supplementary Figure S1B). This confirms that axonal reinnervation is compromised in aged mice.

To understand whether this was due to intrinsic defects in the capacity of PNS neurons to regenerate axons, we used an *in vitro* approach to compare young vs. aged axon growth. We cultured DRG neurons from 2-month and 24-month old mice in parallel then assessed the percent of neurons with outgrowth, mean outgrowth per neuron, and branches per neuron after 16–24 h in culture. Interestingly, there was no significant difference in any metrics we assessed in young axons as compared to aged axons (Supplementary Figures S1C–F), consistent with other findings (Painter et al., 2014; Scheib and Höke, 2016). Together, this suggests that the aged microenvironment plays a role in dysregulated neuro-regenerative responses in aged mice.

### Fewer Macrophages Are Found at the Injury Site of Aged Nerves

There are several potential factors in the aged microenvironment that might account for defects in axonal regeneration, including; defects in fibrosis (Martinod et al., 2017); deficient Schwann cell function (Painter et al., 2014); and shortcomings in the immune system (Montecino-Rodriguez et al., 2013). Given the widespread presence of macrophages at nerve injury sites and the prominent role macrophages play in PNS regeneration (Barrette et al., 2008; Stratton et al., 2018), it is highly likely that these cells are involved. Thus, we first assessed macrophage populations over time in the injured nerves of aged vs. young mice, to establish macrophage dynamics during nerve healing in aging. This revealed that at 3 days following injury, there were 3.6 times fewer macrophages in the aged nerves as compared to the young nerves (Supplementary Figures 2A,B; Day 3 Aged, 37.45 ± 12.56 vs. Day 3 Young, 135.5 ± 28.82) consistent with the findings of other studies (Painter et al., 2014). Intriguingly, macrophage numbers in aged nerves had already returned to values comparable to young nerves by 8 days (Day 8 Aged, 278.1 ± 18.86; Day 8 Young, 222.8 ± 44.56). Given that others have shown that macrophages enhance axonal regeneration (Kigerl et al., 2009), this could suggest that defective regeneration in aging is partially attributed to the absence of macrophages during the critical early-stage post-injury in the aged microenvironment.

### Monocyte Recruitment Is Reduced With Age Due to Defects in PNS Recruitment Signals

**Figure 1:**
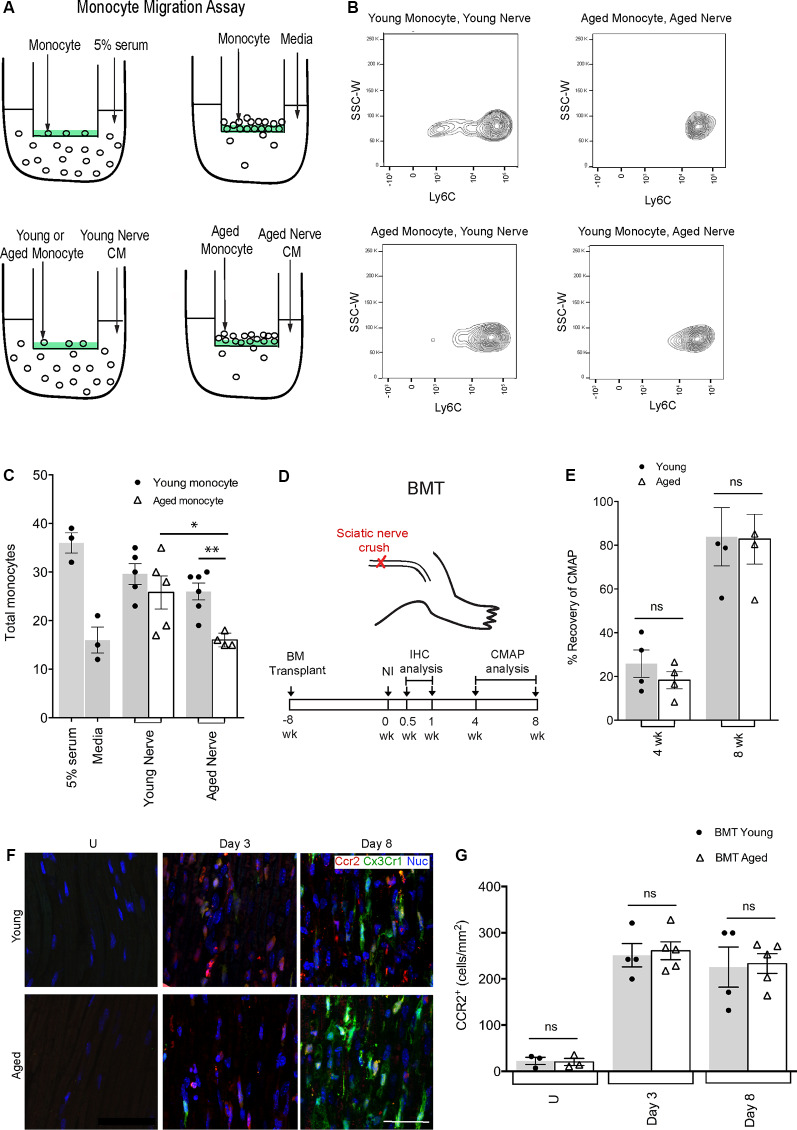
Defects in aged macrophage dynamics can be rescued by providing a young environment. **(A)** Experimental design for monocyte migration assay. Monocytes were placed on a vessel containing a semipermeable membrane, inside a well containing either 5% serum (positive control), media (negative control), young nerve conditioned media (CM) or aged nerve CM. **(B)** Representative flow cytometry plots demonstrating a decrease in LyC6+ monocytes in the condition where aged monocytes were subject to aged CM (Aged Monocyte, Aged Nerve) compared to other groups. This effect was particularly evident in the LyC6^low^ monocyte population. **(C)** Quantification revealed aged monocytes subjected to the aged nerve CM had similar migration as the negative control (media), while young monocytes subjected to young nerve CM had similar values to the positive control (5% serum). Aged monocytes in aged CM showed significantly lower migration than young monocytes in the aged CM. Aged monocytes in the aged CM also showed significantly lower migration than aged monocytes in young CM. *p < 0.05 and **p < 0.02. Error bars indicate ± SEM. **(D)** Experimental design for bone marrow transplant (BMT) experiments. The timeline shows BMT was performed at 8 weeks, sciatic nerve crush injury at 0 weeks, immunohistochemical (IHC) analysis at 0.5 and 1-week post-injury, and compound muscle action potential (CMAP) analysis at 4 and 8 weeks post-injury. **(E)** Quantification of CMAP analysis showing no significant difference in the percent recovery of CMAP amplitude between the young and aged animals at 4 and 8 weeks post-injury, thus illustrating similar functional recovery. *n* = 4 per group; ns, no significance. Error bars indicate ± SEM. **(F,G)** Representative IHC images and supporting quantification showed that at day 3 and 8 post-injury, there was no significant difference in macrophage numbers between young and aged animals. *n* = 4–5 per group; ns, no significance. Error bars indicate ± SEM. Scale bar, 50 μm.

To further our understanding of why there were reduced macrophage numbers in the aged injured nerve, we compared the motility and PNS-specific recruitment signals for young and aged monocytes *in vitro* (Figures 1A–C). We placed young or aged monocytes in a permeable vessel subjected to either young or aged nerve conditioned media (CM). We found that young monocytes were able to migrate similarly towards both young and aged nerve CM suggesting that young monocytes can overcome any age-related defects in recruitment signals from aged nerves (29.60 ± 2.14 and 26.00 ± 1.75, respectively). Aged monocytes however migrated significantly less to aged CM than to young CM (16.00 ± 0.71 and 25.80 ± 3.40, respectively). This suggests that the aged environment, rather than defects in aged monocyte motility/chemoattractant sensing, is necessary for defective recruitment.

### A Young Environment Can Rescue Defects in Aged Macrophage Accumulation and Functional Recovery

To further clarify the environmental influence in regulating aged macrophage dynamics, we performed bone marrow transplants (BMT; Figure 1D). Bone marrow from *Cx3cr1*-*GFP^+^*/*Ccr2*-*RFP^+^* donor mice (either 18–20-month-old or 2-month-old) was transplanted into young recipient C57BL/6 mice. By 8 weeks post-BMT, over 90% of circulating monocytes were *Ccr2*-RFP^+^ and *Cx3cr1*-GFP^+^ and thus derived from aged or young transplanted bone marrow (not shown). We then performed peripheral nerve crush injuries and assessed *Ccr2*-RFP^+^*/Cx3cr1*-GFP^+^ macrophages at 3 and 8-days post-injury (Figures 1F,G). We found no difference in nerve macrophage numbers between the aged or young BMT mice at either time point, suggesting that any defects in macrophage accumulation in aging (Supplementary Figures S2A,B) could be rescued given a more permissive (young animal) environment. Importantly, the percent recovery of CMAPs was also the same for recipients of young or aged bone marrow (Figure 1E). Together these data suggest that aged macrophages are inherently capable of supporting nerve repair if provided appropriate instructions.

### MCP1, a Chemokine Primarily Expressed by Macrophages, Is Downregulated With Advanced Age and Can Significantly Enhance Axonal Growth *in vitro*

**Figure 2:**
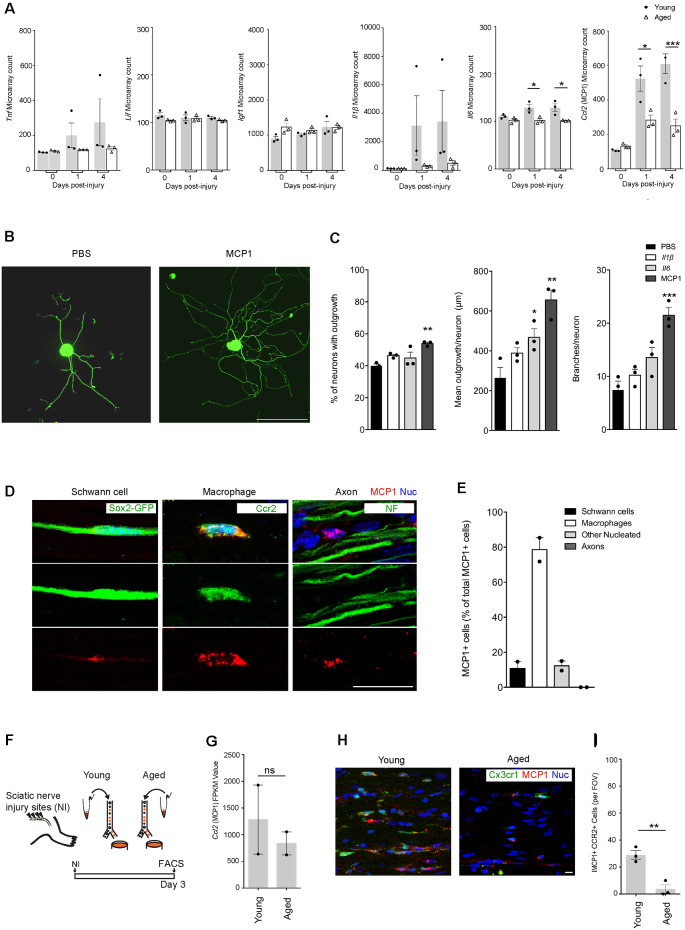
MCP1 positive macrophages are reduced in aging, and MCP1 is a potent promoter of axonal outgrowth. **(A)** Selection of ligands expressed in nerves before and after the injury as per microarray dataset (12). Note the decrease in aged nerves of some (*Il6*, *Ccl2*) but not other (*Igf-1*, *Lif*) cytokines and growth factors known to be expressed by macrophages (19). *p < 0.03 for the *Il6* graph (vs. PBS), *p < 0.05; ***p < 0.0001 for the *MCP1* graph (vs. PBS). **(B,C)** Representative IHC images **(B)** and supporting quantification **(C)** demonstrating that neurite outgrowth was increased in dorsal root ganglion (DRG) neurons cultured *in vitro* with MCP1 vs. PBS, IL1β, and IL6. IL6 showed significant improvement in the mean outgrowth per neuron compared to PBS **(C)**. The percent of neurons with outgrowth, mean outgrowth per neuron, and branches per neuron were all shown to be enhanced with MCP1. **p < 0.007, **p < 0.002 and ***p < 0.0001 (vs. PBS) for graphs 1–3, respectively. Scale bar, 200 μm. **(D,E)** Representative IHC images **(D)** and quantification **(E)** demonstrated that MCP1 is primarily expressed by macrophages (Ccr2, green) in the injured nerve, and not Schwann cells (Sox2-GFP, green), axons (NF, green) and other nucleated cells. Scale bar, 5 μm. **(F)** Experimental design for macrophage RNAseq analysis. Macrophages were FACS collected from young and aged *Cx3cr1*^GFP^/*Ccr2*^ RFP^ mice 3 days after injury. Cells were lysed and sequenced as per the methods section. **(G)** Macrophage RNAseq analysis revealed no significant difference between the *Mcp1* FPKM values in young vs. aged groups. FPKM, Fragments Per Kilobase of transcript per Million mapped reads. ns = no significance. **(H,I)** Representative IHC images **(H)** and supporting quantification **(I)** demonstrated less MCP1*+* macrophages per field of view (FOV) between aged and young injured nerves. **p < 0.005. Scale bar, 10 μm. All error bars indicate ± SEM.

A previous transcriptional profiling study using bulk-RNAseq of injured nerves from 2 and 24-month old mice highlighted age-associated changes in gene expression (Painter et al., 2014). Genes associated with immune signaling were especially intriguing (Stratton et al., 2018). While some genes such as *Tnf, Lif*, and *Igf1* showed no difference with regards to age, *Il1*β, *Il6*, and *Ccl2* (MCP1) were of particular interest as they were found to be downregulated in aging (Figure 2A). To better understand the direct role that Il1β, Il6, and MCP1 may play in regulating axonal regeneration under-aged conditions, we assessed axonal regrowth in aged DRG neurons following their stimulation with these factors *in vitro* (Figures 2B,C). While l1β had no effect, we found that MCP1 consistently increased neurite regrowth; it increased the percentage of neurons with neurites from 39.93 ± 1.13% to 53.91 ± 0.83%, the mean outgrowth per neuron from 264.6 ± 51.94 μm to 655.6 ± 49.99 μm and the number of branches per neuron from 7.46 ± 1.66 to 21.48 ± 1.44 (Figure 2C). Il6 also showed a statistically significant increase in the mean outgrowth per neuron (Figure 2C). Il6 is a known pro-regenerative cytokine, and the modest increase may be explained by its action on initiating regenerative cascades or overriding inhibitory signals (Cao et al., 2006; Pieraut et al., 2011; Cox et al., 2017).

We next assessed the cell types expressing MCP1. As expected from prior genetic analyses (Stratton et al., 2018), our immunofluorescence analysis showed significant colocalization between MCP1+ and CCR2+ macrophages (78.58 ± 6.79% of MCP1 positive cells were macrophages; Figures 2D,E). Quantification also showed MCP1 was expressed by Schwann cells (10.81 ± 3.83%), and other nucleated endoneurial cells (12.26 ± 2.74%), but not axons (0%; Figures 2D,E). Next, to determine if *Ccl2* (MCP1) was differentially expressed between aged vs. young macrophages, we assessed the transcriptional profile of macrophages post-nerve injury from Cx3cr1-GFP/Ccr2-RFP transgenic reporter mice and found no significant difference between groups (Figures 2F,G). However, IHC analysis revealed a marked decrease in the number of MCP1+ CCR2+ macrophages in aged animals as compared to young (Figures 2H,I). This suggests that while *Ccl2 (MCP1)* transcription in macrophages is not dysregulated with aging, the overall numbers of recruited MCP1+ macrophages are diminished with advanced age, likely contributing to a net reduction of *Ccl2 (*MCP1*)* in the aged nerve after injury (Painter et al., 2014).

### Both Pro- and Anti-inflammatory Cytokines Expressed by Macrophages Following Nerve Injury Are Downregulated in Aged Mice Compared to Young Mice

**Figure 3:**
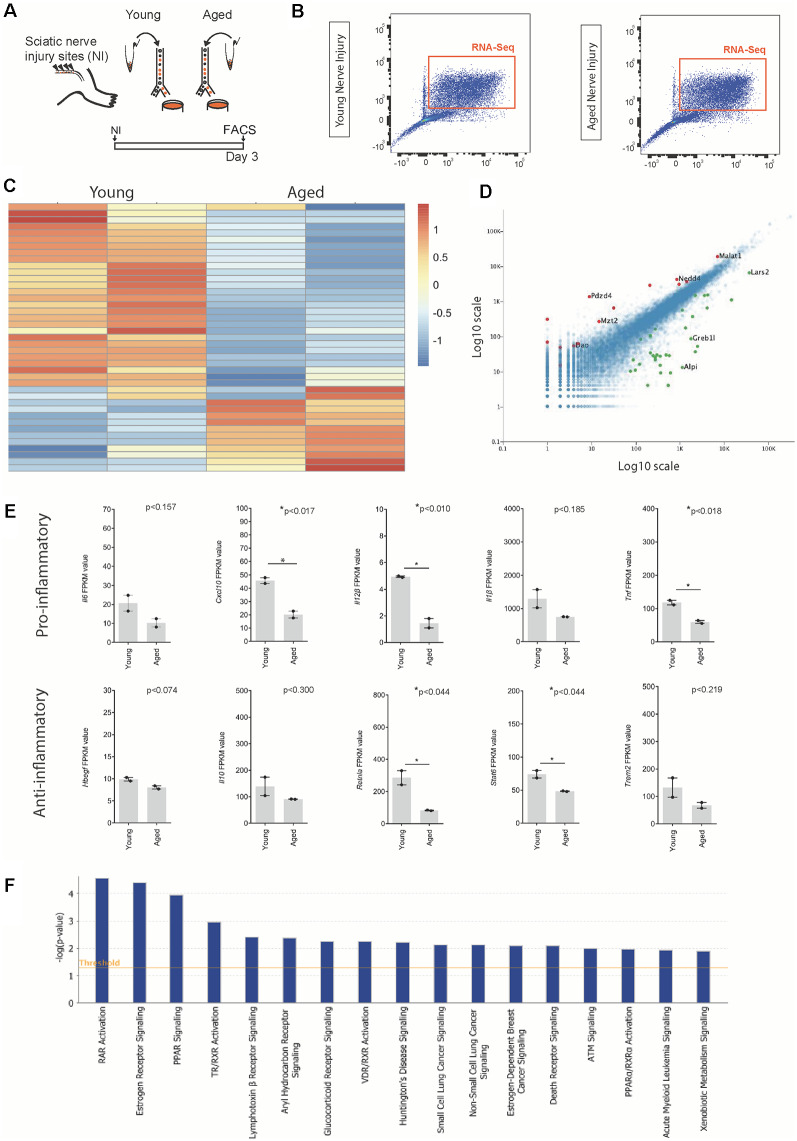
Identification of ligands expressed by macrophages following nerve injury. **(A)** Experimental design for macrophage RNAseq analysis. *Cx3cr1-*GFP^+^* Ccr2-*RFP^+^ double-positive macrophages were FACS collected from young and aged *Cx3cr1*^GFP^/*Ccr2*^ RFP^ mice animals 3 days after injury. Cells were lysed and sequenced as per the methods section. **(B)** FACS plots depicting cell sorting of *Ccr2*-RFP and *Cx3cr1-*GFP positive macrophage cells from the injured nerve. Cells boxed in orange were collected for RNA sequencing. **(C)** Heat map showing the unique expression profile of the top 50 most differentially regulated transcripts between young and aged mice at 3 days post-injury. Red indicates a fold increase while blue indicates a fold decrease. **(D)** Volcano plot demonstrating significantly upregulated (red, +1) and downregulated (green, −1) genes in aged vs. young macrophages. **(E)** FPKM values of common pro-inflammatory (M1) and anti-inflammatory (M2) macrophage identifiers. Error bars indicate ±SEM. **(F)** Top canonical pathways regulated with aging macrophages at day 3 post-injury (threshold cut off 0.05).

To identify other transcriptional differences between aged and young macrophages following nerve injury, we compared the transcriptomes of macrophages from aged and young nerves at day 3 post-injury (Figure 3; young data previously analyzed in (Stratton et al., 2018). Analysis of the top 50 most differentially regulated transcripts revealed a separation in the pattern of gene expression between aged and young macrophages (Figure 3C). The expression of some well-characterized macrophage-associated factors *(Itgam, Mrc1*) but not others (*Ccr2, Cx3cr1*) were downregulated with aging (Figure 3D). In aged macrophages, we also observed suppression of factors typically associated with both a pro-inflammatory macrophage phenotype (*Il6, Cxcl10, Il12β, Il1β, Tnf*) and an anti-inflammatory state (*Trem2, Stat6, Il10, Hbegf, and Retnla*; Figure 3E).

When we assessed the top regulated canonical pathways in aging, we found the three most highly downregulated pathways were; Retinoic acid receptor (RAR) Activation, Estrogen receptor signalling and Peroxisome proliferator-activated receptors (PPAR) Signaling; while the top regulated activation z-scores for cellular functions were; “Organismal death” (Activation Z-score: 2.2; *P*-value: 6.4 × 10–03), “Organization of cytoskeleton” (Activation Z-score: −2.1; *P*-value: 6.7 × 10–04), “Microtubule dynamics” (Activation Z-score: −2.1; *P*-value: 1.6 × 10–03) and “Coordination” (Activation Z-score: −2.2; *P*-value: 8.3 × 10–03). Others have also described age-associated dysregulation of RAR and PPAR signaling in myeloid cells and found that phagocytosis impairment due to a dysregulation in this pathway delayed nervous system regeneration (Natrajan et al., 2015).

## Discussion

To date, numerous studies have outlined the phenomenon of impaired axonal regeneration in aged animals, however, the causes of this from a macrophage perspective have yet to be fully explored. Through observing similar growth rates between young and aged DRG neurons *in vitro*, it was found that the declining regenerative capacity with age could be attributed to the aged microenvironment, which aligns with previously published literature (Kang and Lichtman, 2013; Painter et al., 2014; Scheib and Höke, 2016). In previous experiments, age-mismatch sciatic nerve grafts demonstrated that aged animals could achieve robust functional recovery upon receiving a young nerve graft (Painter et al., 2014; Scheib and Höke, 2016). Conversely, young animals receiving aged nerve grafts displayed similar regeneration to the aged phenotype. We found that aged macrophage numbers can be rescued in young nerve environments and achieve functional recovery similar to young conditions. This provides compelling evidence that macrophage dysregulation is likely one, of several surrounding dysregulated mechanisms, in the aged nerve injury environment. Several factors likely contribute to dysregulated macrophage dynamics. Blood vessel function is impaired, including dysfunction of endothelial, smooth muscle cells, and extracellular matrix vasculature, which could impair infiltration (Rubio-Ruiz et al., 2014). Reduced expression of chemoattractants at the injury site, such as Il6, important for initiating recruitment could also contribute (Tofaris et al., 2002; Painter et al., 2014) as our data suggests. An alteration in macrophage phenotype (Chen et al., 2015), or reduced phagocytic capacity (Natrajan et al., 2015), would also modify macrophage function in a manner which is detrimental to regeneration. And finally, as we have suggested here, a reduction in pro-regenerative macrophage secreted factors, not limited to MCP1, may also contribute.

Previous studies have used high-throughput whole-tissue profiling of immune factors following nerve injury in aged vs. young nerves at acute time points (Painter et al., 2014; Büttner et al., 2018). Given the delay in the accumulation of macrophages with aging, differential expression between groups is difficult to interpret in these studies. Are reductions in immune-associated factors simply due to reductions in infiltrated cell numbers? Or does this reflect a difference in the endogenous capacity of a given macrophage to express genes or proteins of interest? For MCP1, we found that the profile of each individual cell is no different between aged and young macrophages, but there are far fewer macrophages recruited to the injury site in aging which accounts for the reduced overall MCP1 levels in aging conditions (Painter et al., 2014). Employing our macrophage-specific reporter system followed by FACS to prospectively isolate mobilized cells directly from injured nerves enabled us to more directly identify macrophage transcripts, in an unbiased fashion *in vivo*. In support of many conclusions made using bulk tissue analysis approaches, we also observed a decrease in several immune-related genes in macrophages with aging follow acute injury (including *Ilβ, Il6*, and *Cxcl1*). But in contrast to others (Painter et al., 2014), we found that several genes were unchanged in macrophages, including *Ccl11*, *Il16* and *Ccl2*; while others that were upregulated in our dataset were not altered in other datasets (*Cxcl10*, *Il10*, Painter et al., 2014; Büttner et al., 2018). Perhaps this discrepancy reflects changes due to regulation in other cell types, such as the mesenchymal population, or other immune cells which also express several immune-associated genes (Carr et al., 2019) and are sequenced when using whole-tissue techniques. In any case, our data identifies several candidate immunomodulatory factors in macrophages that are regulated with aging within the injured peripheral nerve. This highlights the importance of including population-specific gene expression studies or single-cell studies to better elucidate cell-type-specific transcriptional changes within injured tissues (Stratton et al., 2018; Ydens et al., 2020). Two other studies have used this type of approach to study nerve injury but only in young mice. Together these studies similarly report that *Trem2, Chil3, Arg1*, and several other pro and anti-inflammatory genes are dynamically regulated following injury (Stratton et al., 2018; Ydens et al., 2020). Ydens et al. (2020) also described two steady-state resident macrophage phenotypes and confirmed that the major macrophage type present following injury is the infiltrating macrophage, consistent with the findings reported by fate mapping strategies (Plemel et al., 2020).

We were surprised to find that genes associated with either a classic inflammatory (M1) or anti-inflammatory (M2) macrophage phenotype did not have opposing patterns of expression correlating with aging (Lee et al., 2013; Jablonski et al., 2016). Instead, both gene sets were largely suppressed acutely in aging following nerve injury. There are suggestions that a pro-inflammatory environment is detrimental to neuro-regeneration and an anti-inflammatory environment is beneficial (Mokarram et al., 2012). As an extension, it is reasonable to suggest that a pro-inflammatory environment in aging might be a contributing factor that impairs efficient regeneration, whereas an anti-inflammatory environment would benefit the aging environment (Büttner et al., 2018). Interestingly, both our results and others (Büttner et al., 2018) do not support this suggestion. Rather, both pro- and anti-inflammatory genes are suppressed acutely following nerve injury and largely continue to be dysregulated as time passes, or with macrophage modulation therapies (Büttner et al., 2018). Like us, others have also described age-associated dysregulation of RAR and PPAR signaling, specifically concerning macrophage phagocytosis in the CNS (Natrajan et al., 2015). In aging, it is thought that a reduction in phagocytosis contributes to delayed nervous system regeneration, where defects in retinoic acid signaling underlie this dysfunction (Natrajan et al., 2015). Now that we have identified dysregulated signaling pathways in aging macrophages *in vivo*, future experiments endeavor to manipulate these signatures in nerve injury environments with the hopes of identifying pathways that can be therapeutically modulated to improve regeneration.

Both acute and chronic dysregulation in macrophage dynamics likely contribute to reduced axonal regrowth (Painter et al., 2014; Scheib and Höke, 2016) but we believe the immediate delay in macrophage numbers may play a crucial role in impaired regeneration. This is because, during acute time points, regeneration is usually extremely efficient and axonal regrowth is widespread (McQuarrie et al., 1977; Danielsson et al., 1996; Wang et al., 2015) thus depriving the aged nerve of pro-regenerative macrophages at this critical stage could interfere with this process. Given macrophages express MCP1 at high levels, and MCP1 promotes axonal regrowth, this could be one possible mechanism underlying why axonal regeneration in aging is reduced. MCP1/CCL2 has an affinity for CCR2 and CCR4 receptors, which are both expressed by neurons (Coughlan et al., 2000; Banisadr et al., 2005; Cédile et al., 2017). Kwon et al. (2015) previously showed that an intraganglionic injection of recombinant MCP1 into the L5 DRG of young rats caused significantly greater macrophage accumulation as well as significantly larger neurite outgrowth while injecting other chemokines (CX3CL1 and CCL3) only resulted in macrophage accumulation (Kwon et al., 2015). Also, Niemi et al. (2016) described the effects of MCP1 signaling on axonal growth in young rodents. By infecting neurons with AAV5-CCL2 they found that increased axonal outgrowth was dependent on CCR2 and STAT3 signaling. These findings agree with our data in aged axons, albeit we demonstrate that exogenous application of MCP1 is sufficient to support enhanced axon growth *ex vivo*. We used semi-automated neurite analysis and found an increase in several parameters: mean outgrowth per cell, an increase in the branching of neurites as well as an increase in the percentage of neurons with outgrowth. This was unique to MCP1 given other chemokines such as CCL3 and CX3CL1 had no effect (not shown).

Interestingly, we found the major cell type expressing MCP1 at the site of injury was macrophages, with no detectable levels in axons and substantially less in Schwann cells or other cells within the endoneurial microenvironment. When Kwon and colleagues assessed the ganglion, where the neuronal cell bodies lie, they attributed the major source of MCP1 to be neurons (Kwon et al., 2015). This illustrates the clear regional differences in protein expression apparent within neurons (Matus et al., 1981; Drake and Lasek, 1984). Others have suggested that Schwann cell-derived MCP1 is essential for glia-derived recruitment signals (Subang and Richardson, 2001; Taskinen and Röyttä, 2001; Tofaris et al., 2002). Our characterization of MCP1 expression across various cell types after nerve injury, reveals for the first time that macrophages are the overwhelming source of MCP1 within the endoneurial environment after injury.

MCP1 is a particularly difficult immune-factor to study *in vivo*, partly because it plays a role in both attracting immune cells (Tofaris et al., 2002) and is a growth factor for peripheral axons. Therefore, manipulation of this protein in macrophages *in vivo* would affect its role as a macrophage chemoattractant and as a neurotrophic factor. Dissecting the relative contribution of each role to overall regeneration would be complex. One could imagine the over-expression of MCP1 could result in over-recruitment of macrophages *in vivo*, which may result in neurotoxic effects that negate the neurotrophic benefit (Gensel et al., 2009; Kigerl et al., 2009). This supports our use of an *in vitro* approach, demonstrating directly that MCP1 does have the capacity to enhance axonal regeneration of aged injured nerves.

While our findings indicate that the addition of MCP1 allows for greater axonal regrowth, which provides support for the use of this chemokine/growth factor as a therapeutic agent for neural repair in aged patients, it is important to note that this chemokine/growth factor is also linked to many deleterious health conditions. MCP1 has been shown to have pro-tumorigenic roles that aid in metastasis and has been linked to cardiovascular disease, type 1 diabetes, and obesity (Deshmane et al., 2009; Panee, 2012). For this reason, any future therapeutic strategies must take caution to provide targeted approaches. Further studies assessing how MCP1 can be administered to promote neural repair without causing unintended negative consequences are of great interest.

## Data Availability Statement

The RNAseq data reported in this study has been deposited under GEO submission GSE132882, and portions of this dataset are also found at GSE106927.

## Ethics Statement

The animal study was reviewed and approved by University of Calgary Health Sciences Animal Care Committee, in accordance with the Canadian Council of Animal Care Guidelines.

## Author Contributions

SJ, JS, and JB wrote the manuscript. JS and JB designed the experiments. JS, SE, NR, AH, SJ, and GY performed the experiments and analyzed the data. RM, SE, NR, SJ, JB, and JS read and edited the article. RM and JB supervised all experiments.

## Conflict of Interest

The authors declare that the research was conducted in the absence of any commercial or financial relationships that could be construed as a potential conflict of interest.

## Supplementary Material

FIGURE S1 Aged rodents display reduced recovery post-nerve injury, while aged axons regrow efficiently* in vitro*. **(A)** Experimental design. CMAP analysis was performed in rodents 4 weeks, 6 weeks, and 8 weeks after a sciatic nerve crush injury. **(B)** Quantification of CMAP data showed that aged animals had significantly less % recovery of CMAP amplitude as compared to the young group. *n* = 5–6 per group; **p* < 0.05. Error bars indicate ± SEM. **(C–F)** Representative images and quantification of *in vitro* DRG growth assay demonstrated that neurites from mice aged 2 months and 24 months showed no significant difference in the percent of neurons with outgrowth **(D)** mean outgrowth per neuron **(E)** and branches per neuron **(F)**. *n* = 3 per group. Error bars indicate ± SEM. Scale bar, 500 μm. NI, Nerve injured; CMAP, Compound muscle action potential; DRG, Dorsal root ganglion; ns, no significance.

FIGURE S2 Macrophage response following nerve injury is delayed in aged rodents. **(A,B)** Representative immunohistochemical images demonstrated that following nerve injury, there are reduced macrophage densities at day 3 post-injury in aged rodents compared to young **(A)**. This observation was also supported by quantification **(B)**. *n* = 3–4 per time point; **p* < 0.05. Error bars indicate ±SEM. Scale bar, 50 μm. U, Uninjured.
