# Supplementary figures and images for: Macrophages and Associated Ligands in the Aged Injured Nerve: A Defective Dynamic That Contributes to Reduced Axonal Regrowth
Source: Front Aging Neurosci. 2020 Jun 12;12:174. doi: 10.3389/fnagi.2020.00174 (PMC7304384; doi:10.3389/fnagi.2020.00174)

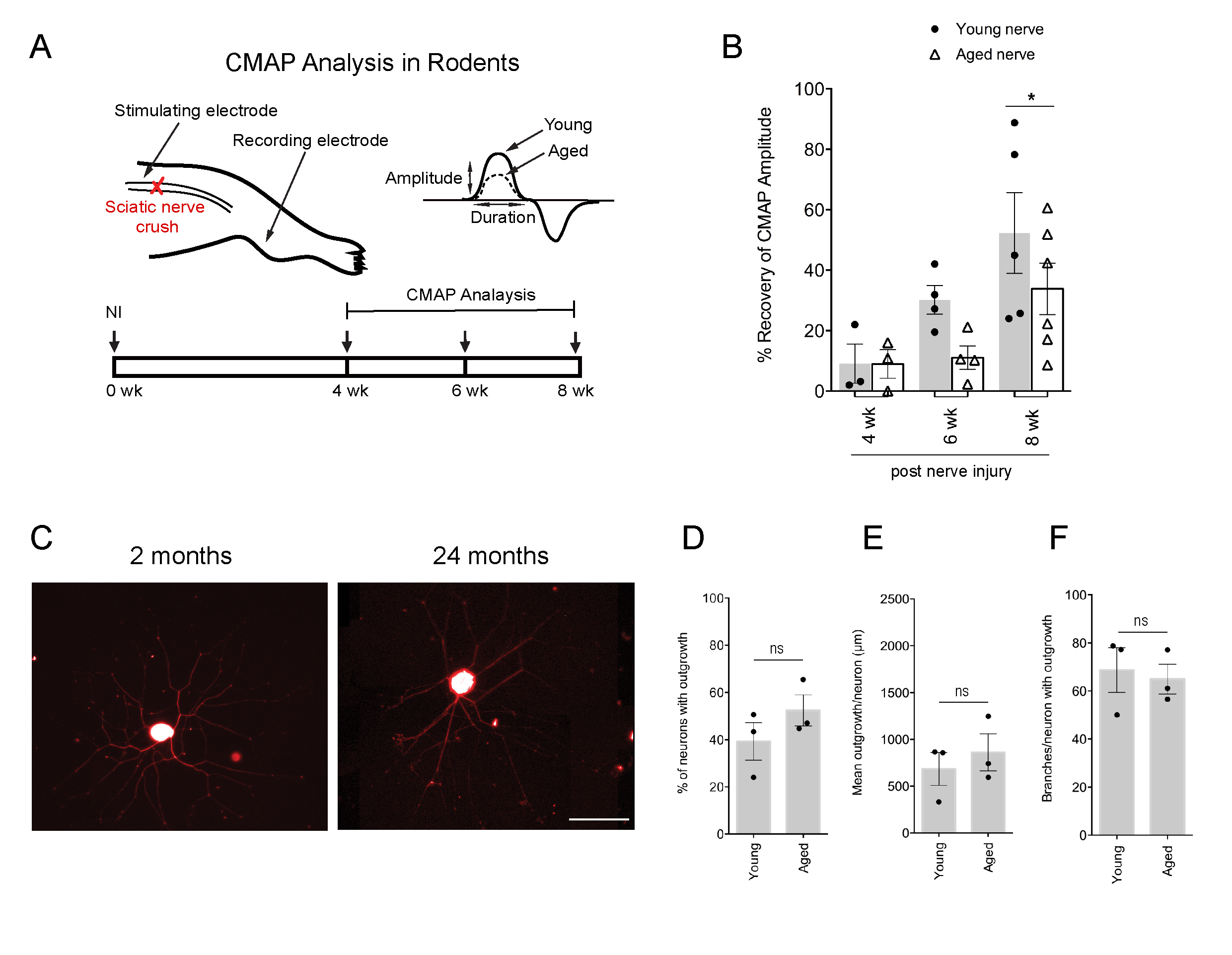

Supplement: FIGURE S1 — Aged rodents display reduced recovery post-nerve injury, while aged axons regrow efficiently in vitro. (A) Experimental design. CMAP analysis was performed in rodents 4 weeks, 6 weeks, and 8 weeks after a sciatic nerve crush injury. (B) Quantification of CMAP data showed that aged animals had significantly less % recovery of CMAP amplitude as compared to the young group. n = 5–6 per group; *p < 0.05. Error bars indicate ± SEM. (C–F) Representative images and quantification of in vitro DRG growth assay demonstrated that neurites from mice aged 2 months and 24 months showed no significant difference in the percent of neurons with outgrowth (D) mean outgrowth per neuron (E) and branches per neuron (F). n = 3 per group. Error bars indicate ± SEM. Scale bar, 500 μm. NI, Nerve injured; CMAP, Compound muscle action potential; DRG, Dorsal root ganglion; ns, no significance. [file Image_1.tif]

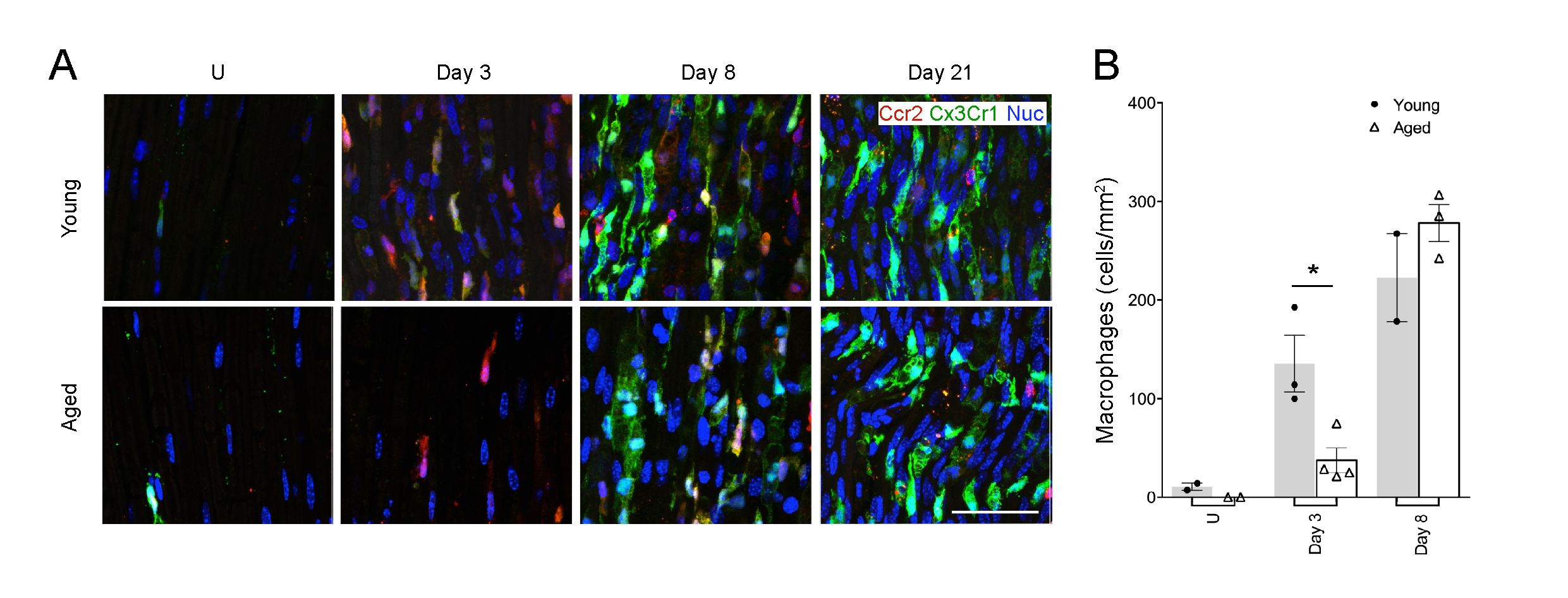

Supplement: FIGURE S2 — Macrophage response following nerve injury is delayed in aged rodents. (A,B) Representative immunohistochemical images demonstrated that following nerve injury, there are reduced macrophage densities at day 3 post-injury in aged rodents compared to young (A). This observation was also supported by quantification (B). n = 3–4 per time point; *p < 0.05. Error bars indicate ±SEM. Scale bar, 50 μm. U, Uninjured. [file Image_2.tif]
